# Psychosocial consequences and early life factors impact on the occurrence of childhood sexual assault among secondary school students in Southern Ethiopia: unmatched case-control study

**DOI:** 10.3389/frph.2024.1369245

**Published:** 2025-03-28

**Authors:** Dagne Deresa Dinagde, Habtamu Wana Wada, Shambel Negesa, Bekam Dibaba Degefa, Gemeda Wakgari Kitil, Gizu Tola Feyisa

**Affiliations:** ^1^ Departments of Midwifery, College of Health Sciences, Mattu University, Mattu, Ethiopia; ^2^ Departments of Midwifery, College of Medicine and Health Sciences, Arba Minch University, Arba Minch, Ethiopia

**Keywords:** abuse, assault, childhood, rape, in Ethiopia

## Abstract

**Background:**

Child sexual assault is a violation of fundamental human rights that leads to various negative consequences, including psychological and physical problems. While it is the least reported and addressed form of violence against schoolgirls in Ethiopia, it is a public health issue that affects millions of people globally each year. Thus, this study aims to provide information on the early life factors impact on the occurrence of childhood sexual assault among secondary school students.

**Methods:**

An institution unmatched case-control study was conducted in Arba Minch Zuria district among high school female students attending regular education from March 20, 2023 to May 20, 2023. The data were collected using structured, pretested self-administered questionnaire in all school in Arba Minch Zuria Woreda. Reports of schoolgirls being sexually assaulted were first obtained from the district police office. According to these reports, there were seventy-five (75) cases where the girls were attending or had attended the mentioned high school. In addition, female control students were randomly selected from non-case students. Odds ratio with 95% CI was used as a measure of association, and variables with a *p*-value of ≤0.05 were considered to be statistically significant.

**Results:**

Multivariate logistic regression analysis was used to identify significant factors. Accordingly factors such as had consumed alcohol (AOR = 4.0, 95CI: 1.68–9.70), living with non-biological parents (AOR = 7.49, 95CI: 2.72–13.65) and living alone (AOR = 4.6, 95CI: 1.49–14.41), being street food vendors (AOR = 4.5, 95CI: 1.48–13.70) and visiting library at improper time (AOR = 5.0, 95CI: 1.87–13.47) were significantly associated with childhood sexual assault.

**Conclusion:**

Had consumed alcohol, living with non-biological parents and alone, being street food vendors and improper library time were identified as significant determinants of child sexual assault (CSA) among students included in this study. Thus, high schools and families with children should collaborate to develop a plan to reduce sexual abuse that violates children rights. Families should oversee their children to help prevent substance use during their school years. Additionally, parents should remain close to their children, as distancing can lead to exposure to risky relationships.

## Introduction

Child sexual abuse (CSA) is the term used to describe when a child (under the age of eighteen) is involved in sexual activity for which he or she is not developmentally ready to give informed consent, does not fully understand, is unable to give consent for, or that violates legal requirements or social taboos (1). It is illegal to exploit children for an adult's sexual gratification, and Child sexual abuse is among the most undisclosed problem and least addressed forms of child abuse worldwide (2). Sexual abuse is a widespread violation of human rights that is now considered a public health priority. It affects individuals of all genders, sexual orientations, and ages in every community, but in both industrialized and developing nations, the majority of victims are children (3).

The two primary categories of child sexual abuse that are typically distinguished are contact and non-contact child sexual abuse. Intercourse that involves penetration (like oral and vaginal sex) is considered abuse of contacts. Non-contact activities include coercing or threatening children to watch or produce porn, encouraging kids to act or behave inappropriately in sexual situations, and grooming kids to commit sexual abuse (4).

One out of twenty adolescent girls worldwide reported experiencing coercion during their first Sexual experience. Sometimes there is more overt aggressiveness involved when sexual assault (rape) happens on or around school premises. Thus, globally, the prevalence of CSA among adolescent girls was estimated to be 19.7% (5). Asia and sub-Saharan Africa had greater rates of childhood sexual abuse (23.9% and 34.4%, respectively) than did Europe and America (9.2% and 10.1%) (6, 7). In Sub-Saharan Africa (SSA), sexual abuse of victims often occurs at the hands of family members, neighbors, and acquaintances. A local survey revealed that 34.4% of female respondents reported experiencing sexual abuse during childhood (6). Research among female students in southern Nigeria found that 26% had faced similar experiences (8) and South Africa has one of the highest rates of sexual assault globally, particularly affecting teenage girls aged 12–17, with a vulnerability rate of 39% (9).

In the context of Ethiopian high schools, studies have been done on the magnitude of CSA in Butajira, which was 32.8% (10), in Gandhi Memorial Hospital in Addis Ababa, which was 42.7% (11), in Bahir Dar, which was 37.3% (12), and in Dire Dawa, which was 48.9% (3). According to a WHO report, in many underdeveloped nations, including Ethiopia, CSA is the least recorded form of violence, with barely one out of ten instances being reported (13).

Psychological reactions such as dread, anger, fury, guilt, and/or disbelief are frequently experienced by victims of child sexual abuse (14). Consequently, individuals often experience the symptoms such as post-traumatic syndrome disorder (PTSD), such as the burden of STDs, HIV/AIDS, unintended pregnancy, unsafe abortion, crying, and bleeding (15). Worldwide, there are thought to be 74 million unintended pregnancies annually, which leads to 25 million unsafe abortions and 47,000 maternal deaths (16). But at some time in their lives, 2.9 million American women (2.4%) become pregnant as a result of rape (17).

Children with disabilities or in residential care were particularly vulnerable to these kinds of abuses. When it comes to disabilities, children with mental or intellectual impairments are more vulnerable than those with other kinds (4), poor governance, culture, weak rule of law, unemployment, social and gender norms, gender inequality, low income, restricted educational opportunities, and the absence of one or both parents are all highly associated with sexual abuse (6, 8). Research indicates that children mental health issues significantly influence long-term economic consequences more than childhood physical health issues (18).

Ethiopia signed the United Nations Convention on the Rights of the Child (UNCRC) in 1991. Since then, the government has launched numerous programs to ensure the advancement and defense of children's rights and welfare. Rape or other abuse of a girls and boys between the ages of 13 and 18 by a member of the opposite sex is considered an aggravating circumstance and is punishable by up to 20 years in jail, as per the 2004 Ethiopian modified law [Article 623(2)(a)/2004] (19).

Despite the fact that the occurrence of child sexual abuse is influenced by various factors such as measurement issues, cultural context, complex interactions, sample size and diversity, and temporal factors there have been few studies on the prevalence and severity of child sexual abuse among high school students in Ethiopia. Additionally, little research has explored the early factors that contribute to these events. Therefore, studying child sexual abuse is crucial to developing evidence-based interventions, and thus, this study was conducted to assess the early life factors that are associated with the occurrence of child sexual abuse (CSA) among female high school students in Arba Minch Zuria Woreda, Southern Ethiopia, in 2023.

## Methods and materials

### Study design and setting

An institution-case-control study was conducted in Arba Minch Zuria district among high school female students attending regular education from March 20, 2023 to May 20, 2023. The woreda is situated around Arba Minch Town, the capital of the South Nationalities, Nations, and Peoples (Debub) Regional State. Hawassa, the Sidama region's commercial and administrative hub, is 275 km away, and Addis Ababa, the capital of Ethiopia, is 505 km South-West. In 2023, there were roughly 129,666 people living there. Located in the Gamo zone, it is one of 19 woredas with seven high schools. Located in the Gamo zone, it is one of 19 woredas with seven high schools offering both regular and irregular classes. Of the total number of students enrolled in all high schools, 1,639 were female.

### Study participants

The study participants were female high school students randomly chosen from the source population who met the inclusion and exclusion criteria. The source population for this study consisted of all female high school students enrolled in their education during the academic year 2022/2023.

### Case definition and sample size determination

An institutional (school) based unmatched case control study design was conducted. Reports of schoolgirls having been sexually assaulted were first obtained from the Arba Minch Zuria woreda which provide both day and night education program, Southern Ethiopia. Reports of schoolgirls being sexually assaulted were first obtained from the Arba Minch Zuria district police office. According to this report, there were seventy-five (75) cases who were attending or had attended the mentioned high school. A total of 150 female control students were also randomly selected from non-case students. Then, all cases were interviewed one after the other through home visits for those who had stopped attending school, or one-on-one interactions with school administrators at break time for those currently attending school.

### Eligibility criteria

Students who were critically sick or who attended classes on the weekends were excluded from the study due to the challenges in collecting the required data. Individuals who met the eligibility criteria but refused to give consent to participate in the research were also not included.

### Data collection procedures and data quality

Before the two weeks of actual data collection, 5% of the female students at Chencha Woreda High School completed pretests of the questionnaires. In response, adjustments were made to the instrument, clarity, and ambiguity of the language tool. Five female teachers were chosen to act as data collectors and supervisors due to their previous experience in data collection and proficiency in local languages. They received two days of continuous training on the study's objectives, data collection tools, and techniques. Data was collected by self-administered questionnaires at break time in morning and afternoon without affecting teaching leaching learning processes. To maintain privacy, a separate space was prepared in advance for students, with data collectors and facilitators assigned to each room. The questionnaire should be filled out and placed on the designated table, but participants were instructed not to write their names or any other identifying information on it. As soon as the data was obtained, the supervisors and investigator checked it for accuracy, clarity, and consistency.

### Statistical analysis

Data was collected by self-administered questionnaires and the coded, modified and input into the Epidata version 4.6 software, the data was ready for analysis. Descriptive statistics, which comprised relevant features including frequencies, proportions, and summary statistics, were used to characterize the study population. The association between the dependent and independent variables was evaluated using binary logistic regression. Multi-collinearity and the model's goodness of fit were checked using the VIF (<10) and Hosmer-Lemeshow tests (>0.05), respectively. Accordingly, any variable having *p* ≤ 0.25 was considered a candidate variable for multivariable analysis and entered into a multivariable logistic regression model using the backward elimination stepwise likelihood ratio method. All tests were two-sided, and *p* < 0.05 was considered statistically significant. The results were reported as the odds ratio (OR) and 95% confidence interval (CI). A *p*-value of less than 0.05 was chosen as the level for statistical significance.

### Variables measurement

The outcome variable of this study is the determinants of childhood sexual assault, which includes six items (components) that were extracted and adapted from previously published research (Table 1).

**Table 1:** Questions and responses about child sexual abuse.

Components of childhood sexual abuse and questions	Response	Operational definition
Have you experienced to any form of childhood sexual abuse?	Participants responded with “Yes” or “No” to the questions, and if they answered “Yes,” they were asked to specify the type of sexual abuse they had experienced. 1. Involuntary kissing Forced to look at sexual 1. activities 2. Encourged to behave sexually 3. Unwellcome touch 4. Verbal harassment 5. Rape	The participants were reported to have experienced any form of the listed abuse, coding their responses as “Yes” for those who did and “No” for those who did not (20).
Who was the person that inflicted the abuse on you?	They provided response in the form of ’ 1. Family member 2. Schoomates 3. Teachers 4. Boyfriends 5. Neighbours 6. Strangers/unrecognized person’	The items were recorded starting from 1 to 6 making easy for analysis.
Health consequences after the event	Responses included 1’physical injury’, 2“peer-rejection”, 3’ low-self-esteem’, 4’ stop learning’, 5’unwanted pregnancy, 6’ abortion’, 7'STIs’, and 8’vaginal bleeding’	The items were recorded starting from 1 to 8 making easy for analysis.
The physical forms of sexual abuse:	Responses included 1 “genital contact’, 2“fondling”, and 3 “rape”.	The item was recorded as 0 “NO” if participant never exposed and 1 “Yes” if had history of exposure (3, 21).
Non-physical forms of sexual abuse	Responses included 1 “plain talk about sex”, 2 “watch sexual acts” and 3’ other indecent exposure’	The item was recorded as 0 “NO” if participant never exposed and 1 “Yes” if had history of exposure.

### Key independent variables

#### Having boyfriend

This variable is nominal and has been associated with child sexual abuse in various studies (3, 8, 14). In this study, participants who had boyfriends were coded as 1 “Yes” while those who did not were coded as 0 “No”.

#### Parental conflict

High levels of conflict create a stressful environment that can lead to poor parental mental health and impaired judgment, making it easier for abuse to occur (22, 23). According to this study, participants who reported experiencing any form of parental conflict were coded as 1 “Yes,” while those who did not experience such conflict were coded as 0 “No.”

#### Open discussion with family

Clearly, this variable is nominal and has shown a significant association with the occurrence of child sexual assault (6, 15, 24, 25). It was coded as 1 “Yes” for respondents who felt comfortable discussing reproductive health issues within the family, and 0 “No” for those who were concerned about such discussions.

#### History of sexual intercourse

Respondents with a history of sexual practices were coded as 1 “Yes,” while those without such a history were coded as 0 “No.”

#### Substance abuse

Is the use of alcohol, chat, and/or cigarette in quantities or ways that are harmful to the user or others. Participants who were found to be using substances were assigned a code of 1 for “Yes,” while those who were not using substances received a code of 0 for “No.” (26).

## Results

### Characteristics of the study population

A total of 225 participants (75 cases and 150 controls) were included in the study, for a response rate of 100%. The mean age of the participants was 17.26 years (SD ± 1.54) with a minimum and maximum age of 14 and 22, respectively. Participants who were over 18 were asked only about their exposure to CSA that occurred before they turned 18. The majority of the students, 77.3% and 78.7% among cases and controls, respectively, came from rural areas. One-tenth of the participants, 22 (9.8%), were married; 9.3% of the cases and 10% of the controls. The majority of participants, 66.7% vs. 78.7%, among cases and controls, respectively, had both biological parents. The majority, 57.3% vs. 72%, of participants among cases and controls, respectively, were Gamo in ethnicity, while 48% of mothers among cases and 51.3% among controls were Protestant in terms of their religion.

Among the participants in the cases and the controls, 33.3% and 40.7%, respectively, their father had been working on daily labor. While about 61.3% and 56.7% of the cases and controls were living in families of more than five, 81.3% of the families of the participants among the cases and 88% of the participants among the controls were earning less than 1,317 ETB (Table 2).

**Table 2:** Socio-demographic characteristics of study population, 2023 (*N* = 225).

Variables	Categories	Case (*n* = 75)	Control (*n* = 150)
Age	≤15	10 (13.3%)	29 (19.3%)
	16–17	36 (48%)	58 (38.7%)
	18–19	23 (30.7%)	57 (38%)
	>=20	6 (8%)	6 (4%)
Marital status	Unmarried	68 (90.7%)	135 (90%)
	Married	7 (9.3%)	15 (10%)
Residence	Urban	16 (21.3%)	34 (22.7%)
	Rural	59 (78.7%)	116 (81.7)
Religion	Orthodox	23 (30.7%)	34 (22.7%)
	Muslim	11 (14.7%)	32 (21.3%)
	Protestant	36 (48%)	77 (51.3%)
	Other	5 (6.5%)	7 (4.7%)
Ethnic	Gamo	43 (57.3%)	108 (72.0%)
	Wolaita	12 (16.0%)	14 (9.3%)
	Amhara	12 (16.0%)	23 (15.3%)
	Other*	8 (10.7%)	5 (3.3%)
Father's occupation	Employed	15 (20%)	24 (16%)
	Daily worker	14 (18.7%)	41 (27.3%)
	Merchant	21 (28%)	24 (16%)
	Farmer	25 (33.3%)	61 (40.7%)
Father's education	Unable to read and write	12 (19.4%)	16 (12.8%)
	Primary	34 (54.8%)	61 (48.8%)
	Secondary	16 (25.8%)	48 (38.4%)
Mother's education	Unable to read and write	16 (22.2%)	21 (14.9%)
	Primary	43 (59.7%)	82 (58.2%)
	Secondary	13 (18.1%)	38 (27%)
Family size	Less than 5	29 (38.7%)	65 (43.3%)
	5 & More than 5	46 (61.3%)	85 (56.7%)
Family monthly income	< 1,317 ETB	14 (18.7%)	18 (12%)
	≥ 1,317 ETB	61 (81.3%)	18 (12%)

* Others ethnic Gurage and omo.

### Behavioral characteristics of participants and their family

Only 26.7% of both cases (16%) and controls (32%) slept with their mothers. The majority of the participants, 77.3% vs. 63.3%, had a boyfriend among the cases and control, respectively. Only 45.3% of the participants had an open discussion on sexual and reproductive health issues. About 27.6% of participants’ families (38.7% vs. 22.7%) among the cases and controls used substances like alcohol and chewing or smoking. The majority of participants started sexual practices between the ages of 14 and 16. The largest percentage, 40% vs. 26.7%, of participants among cases and controls were working with street food vendors, while 52% of participants among cases and 29.3% among controls claimed improper library arrangements (Table 3).

**Table 3:** Behavioral characteristics of participants and family of study population, 2023 (*N* = 225).

Variables	Categories	Case (*N* = 75)	Control (*N* = 150)
Co-sleeping	Mother	12 (16%)	48 (32%)
	Sister	34 (45.3%)	73 (48.7%)
	Brother/s	2 (2.7%)	8 (5.3%)
	Alone	27 (36%)	21 (14%)
Parental conflict	Yes	29 (38.7%)	34 (22.7%)
	No	46 (61.3%)	116 (77.3%)
Family substance user	Yes	25 (33.3%)	37 (24.7%)
	No	50 (66.7%)	113 (75.3%)
Have boyfriend	Yes	58 (77.3%)	95 (63.3%)
	No	17 (22.7%)	55 (36.7%)
Open discussion with family	Yes	9 (12%)	93 (62%)
	No	66 (88%)	57 (38%)
Drinking alcohol	Yes	45 (60%)	29 (19.3%)
	No	30 (40%)	121 (80.7%)
Chewing chat	Yes	16 (21.3%)	14 (9.3%0
	No	59 (78.7%)	136 (90.7%)
Age of sexual intercourse	14–16	24 (49%)	38 (55.9%)
	17–19	25 (51%)	30 (44.1%)
Economical supported by	Parental	69 (92%)	144 (96%)
	Non -parental	6 (8%)	6 (4%)
Had been living with	Biological parents	14 (18.7%)	101 (67.3%)
	Non- biological parents	37 (49.3%)	32 (21.3%)
	Alone	24 (32%)	17 (11.3%)
Being street food vendors	Yes	30 (40%)	40 (26.7%)
	No	45 (60%)	110 (73.3%)
Improper library time	Yes	39 (52%)	44 (29.3%)
	No	36 (48%)	106 (69.7%)

### Characteristics of the sexual assault

Most of the students (cases) were assaulted by non-relatives such as neighbors (26.7%), boyfriends (25.3%), and schoolmates (21.3%). About 17.3% of the participants did not know the perpetrator (strangers). Just 7 (9%) of the offenders were detained, placed under arrest, and brought to court. Regarding the place of assault, the majority of 24 (32%) of the students were assaulted at their own home, and 18 (24%) raped on the street or in the field (undefined area). The Among 75 rape survivors, only 4 (5.3%) were assaulted by family members.

### Psychosocial and health consequences of sexual assault (rape)

**Figure 1:**
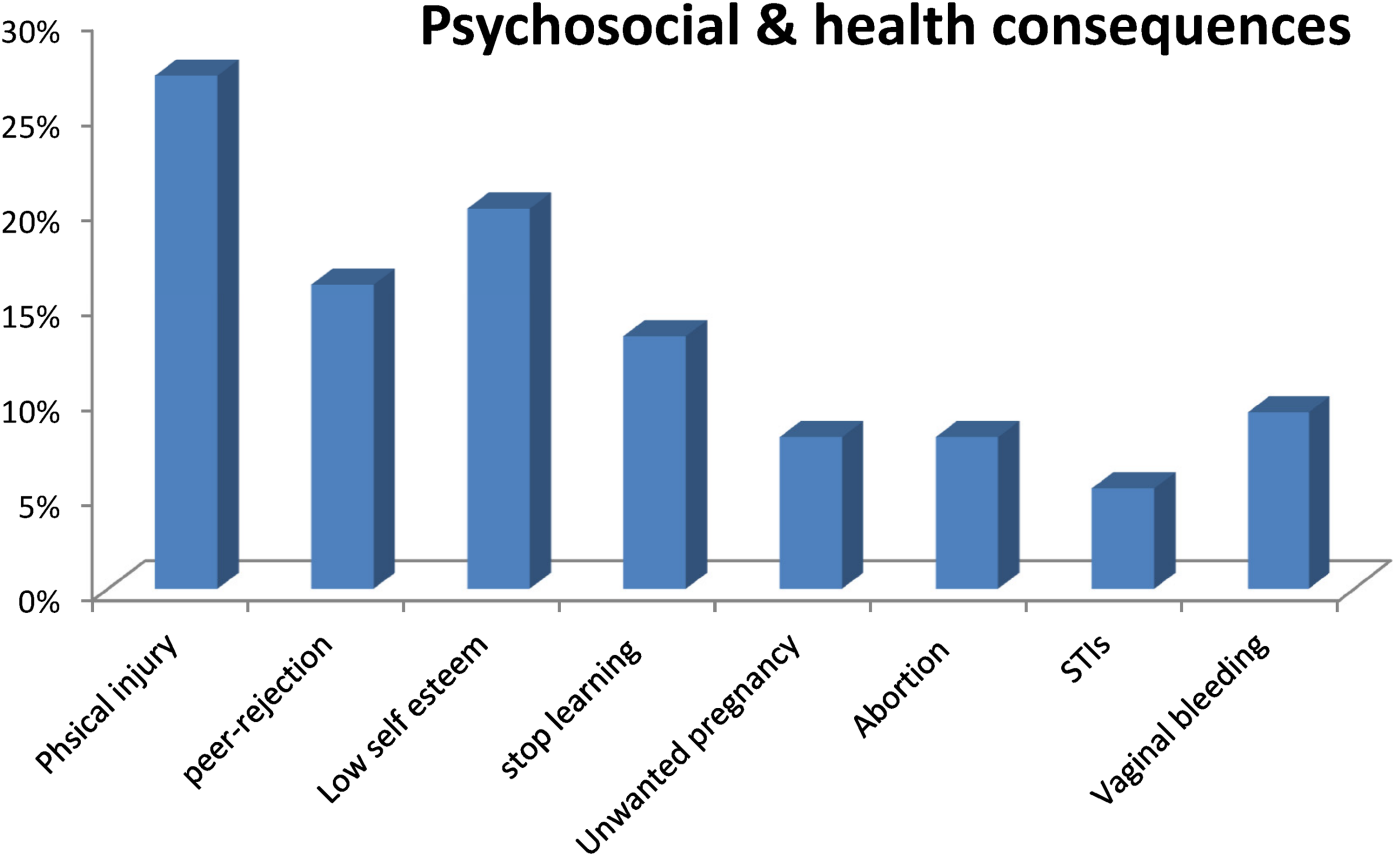
Shows psychosocial and health consequences of sexual assault (rape) among secondary school girls in Arba Minch Zuria woreda, southern Ethiopia, 2023 (*N* = 225).

Among the 75 cases adolescents who reported rape, more than half 40 (53.3%) had visited the clinic and received post exposure prophylaxis for pregnancy and infectious disease. Twenty (26.7%) of the respondents reported having suffered a physical harm during the incident. Twelve respondents (26%) reported that they ended their relationships with both their boyfriend and girlfriend following the rape, and nearly one-fifth (20%) felt they had lost confidence in themselves compared to before the incident (low self-esteem). Additionally, around ten students (13.3%) intended to stop their studies for one to two years due to feelings of humiliation, guilt, and fear. And nearly one-fourth of participants reported become substance users after the assault (Figure 1).

### Factors associated with sexual assault

In the bi-variable logistic regression, ten variables: presence of both parents, parental conflict, having a boyfriend, having a history of sexual intercourse, open discussion with parents, history of alcohol drinking, living with non-biological parents, being street food vendors, and improper library arrangements were statistically significantly associated with childhood sexual assault at a *p*-value of 0.25. All these variables were included in the multivariable logistic regression model, but other variables that had a *p*-value greater than 0.25 in this model were not included in the multivariable regression model.

In the multivariate logistic regression model, four independent variables, namely, having ever drunk alcohol, with whom they had been living, being street food vendors, and improper library time arrangements, were found to be statistically significantly associated with life-time childhood assault at a 95% confidence level and a *p*-value of less than 0.05.

The study showed that students who had been using alcohol before incident were about four times more likely to experience childhood assault compared to students who did not use alcohol (AOR = 4.0, 95CI: 1.68–9.70). students who had been living with non-biological parents and alone were about seven and five times more likely to be exposed to sexual assault as compared to those who had been living with their biological parents (AOR = 7.49, 95CI: 2.72–13.65) and (AOR = 4.6, 95CI: 1.49–14.41), respectively. Similarly, participants who had been working as street food vendors (selling) were five times more likely to have experienced sexual assault compared to those who did not participate in this work (AOR = 4.5, 95CI: 1.48–13.70). Likewise, the odds of childhood sexual assault among students exposed to improper library time in school compared to their counterparts were fivefold (AOR = 5.0, 95CI: 1.87–13.47) (Table 4).

**Table 4:** Factor associated with CSA in multivariable logistic regression analysis for secondary school female students, Arba Minch Zuria Woreda, Ethiopia 2023 (*N* = 225).

Variables	Categories	Cases	Control	COR (95% CI)	AOR (95% CI)	*P*-value
		*N* (75)	*N* (150)			
Presence of Both parents	Yes	50	118	1	1	–
	No	25	32	1.84 (.99–3.42)	2.48 (.91–6.77)	0.075
Parental conflict	Yes	29	34	2.15 (1.18–3.92)		
	No	46	116	1	1	
Boy friend	Yes	58	95	1.97 (1.04–3.73)	1.40 (.48–4.06)	0.512
	No	17	55	1	1	–
Had history of sexual intercourse	Yes	49	68	2.27 (1.28–4.04)	1.95 (.75–5.10)	0.175
	No	26	82	1	1	–
Open discussion with family	Yes	9	93	1	1	–
	No	66	57	12 (5.53–25.85)	2.04 (.75–5.53)	0.175
Had ever drunk alcohol	Yes	45	29	6.3 (3.38–11.57)	4.0 (1.68–9.70)	0.002*
	No	30	121	1	1	–
Whom with had been living	Biological parent	14	101	1	1	–
	Non- biological parents	37	32	8.3 (4.01–17.35)	7.49 (2.72–13.65)	0.000*
	Alone	24	17	10.2 (4.41–23.5)	4.6 (1.49–14.41)	0.008*
Had ever chewed chat	Yes	16	14	2.63 (1.20–5.75)	.65 (.17–2.39)	0.521
	No	59	136	1	1	–
Being street food vendors	Yes	30	40	1.8 (1.02–3.29)	4.5 (1.48–13.70)	0.008*
	No	45	110	1	1	–
Improper library time arrangement	Yes	39	44	2.6 (1.47–6.42)	5.0 (1.87–13.47)	0.001*
	No	36	106	1	1	–

* *P* < 0.05 or significant.

## Discussion

This study indicated that 80% of students have experienced at least some form of psychosocial consequences. Among the cases, only half of them had visited the clinic and received post-exposure prophylaxis for pregnancy and infection. Physical harm is the most common consequence of child sexual assault in this study, which covers about 27%. Acts such as slapping, cutting, and genital tearing are examples of physical injury. The survivors or victims may have to deal with behavioral aggression, a sense of unforgettably high sin, and the accommodation of bacterial infection through cutting and, in rare cases, fistula and inconsistence. This justification was supported by different studies and reports conducted worldwide (22, 27–29). Others participants have reported complain of emotional disorders like, peer-rejection, low self-esteem and substance user after then.

Students who had been using alcohol were about four times more likely to experience childhood rape (assault) compared to students who did not use alcohol. On another hand, respondents who had history of alcohol consumption were at higher risk of experiencing sexual assault than their counterparts. This finding is similar with studies conducted in united states (30), Columbia (31) and Dire Dawa, Ethiopia (3). This could be explained by the fact that drinking reduces one's capacity to make thoughtful decisions about relationships pertaining to their sexual and reproductive health. Furthermore, alcohol can occasionally make one depressed and lead them to form indiscriminate relationships with even complete strangers (someone they had never met before).

In this study, child sexual assault was less common (18.7%) among respondents living with their biological parents. Thus, pupils who had been living with non-biological parents and alone were roughly seven and five times more likely to be subjected to sexual assault as compared to those who had been living with their biological parents. This might be due to the parents are more concerned for their daughter than their friends and being alone, this may help reduce the likelihood that they will experience sexual abuse. This finding was consistent with study from third National Incidence Study of Child Abuse in Washington which stated that Children who live with a non-biological parents and that has a live-in partner were at the highest risk: they are 20 times more likely to be victims of child sexual abuse than children living with both biological parents (32).

Participants working as street food sellers were five times more likely to have experienced sexual assault compared to those who did not participate in this work. Because they live and work on the streets all night, street children are vulnerable to acts of violence in the community. Many African cultural settings have a difficult cultural foundation that accepts violence against children. In certain communities, traditional beliefs may condone physical punishment or other forms of violence as acceptable disciplinary measures. Cultural practices, such as initiation rites or certain rituals, may inadvertently inflict harm on children, often justified by longstanding customs. Certain regulations by the government ignore the suffering that these children endure (23, 33, 34).

The data indicates a concerning correlation between improper library time arrangements and the prevalence of child sexual assault. Specifically, 52% of respondents who reported negative reactions to library time arrangements had experienced such assaults. This suggests that students who face inadequate or poorly structured library time are significantly more vulnerable to sexual violence. This may be warranted since students who return to the library improperly may depart at an inappropriate time, perhaps at night, when they are more likely to be sexually assaulted by someone on the street. This is clear from the fact that 24% of survivors reported being raped in a field or on the street.

### Implication of the study

Generally, this study suggests that policymakers should modify the laws regarding illegal acts committed against children, even though there isn't a 100% safe place to prevent juvenile sexual assault. In addition, parents should carefully monitor their school-age children if they read this study. This work serves as a foundation (cornerstone) for further research on the subject of childhood rape by other scholars.

### Strength and limitation of the study

The nature of this study design allows for the assessment of temporal causality between exposure and the problem (disease). However, given the sensitive nature of the study, which addresses personal issues related to sexuality, there may be underreporting of sexual abuse experiences. Therefore, the findings should be interpreted in light of these limitations.

## Conclusion

The study found that several significant factors contribute to child sexual assault (CSA) among students, including alcohol consumption, living with non-biological parents, living alone, working as street food vendors, and negative experiences related to library time. In this study, physical injury, peer-rejection, low self-esteem and unintended pregnancies were the most often seen psychosocial and health consequences of sexual assault on victims. Thus, high schools and families with children, especially those with children who live away from home, should collaborate to develop a plan to reduce sexual abuse that violates human rights while highlighting the above reasons. Finally, the study recommends that future researchers include boys in their investigations of child sexual abuse, as evidence shows that abuse of boys is increasingly reported across various societies.

## Data Availability

The original contributions presented in the study are included in the article/Supplementary Material, further inquiries can be directed to the corresponding author.
